# Supplementary material for: Direct Photochemical Synthesis of Substituted Benzo[b]fluorenes
Source: Org Lett. 2024 Nov 25;26(48):10364–8. doi: 10.1021/acs.orglett.4c03978 (PMC11629382; doi:10.1021/acs.orglett.4c03978)
Supplement: Supplementary file 2 — ol4c03978_si_002.zip [file ol4c03978_si_002.zip › FID for Publication/5s/20240724_RC-2-C-5-132A_01/plots/RC-2-C-5-132A_PROTON_20240724_01_plot01.pdf]

Sample Name:
RC-2-C-5-132A
Data Collected on:
localhost.localdomain-vnmrs500
Archive directory:
/home/data/MBaumann/cscb500/RuairiCrawford/2024
Sample directory:
20240724_RC-2-C-5-132A_01
FidFile: RC-2-C-5-132A_PROTON_20240724_01

Pulse Sequence: PROTON (s2pul)
Solvent: acetone
Data collected on: Jul 24 2024

Temp. 25.0 C / 298.1 K
Sample #4, Operator: MBaumann

Relax. delay 1.000 sec
Pulse 45.0 degrees
Acq. time 2.045 sec
Width 8012.8 Hz
8 repetitions
OBSERVE H1, 499.7218396 MHz
DATA PROCESSING
FT size 32768
Total time 1 min 24 sec

OpenVnmrJ

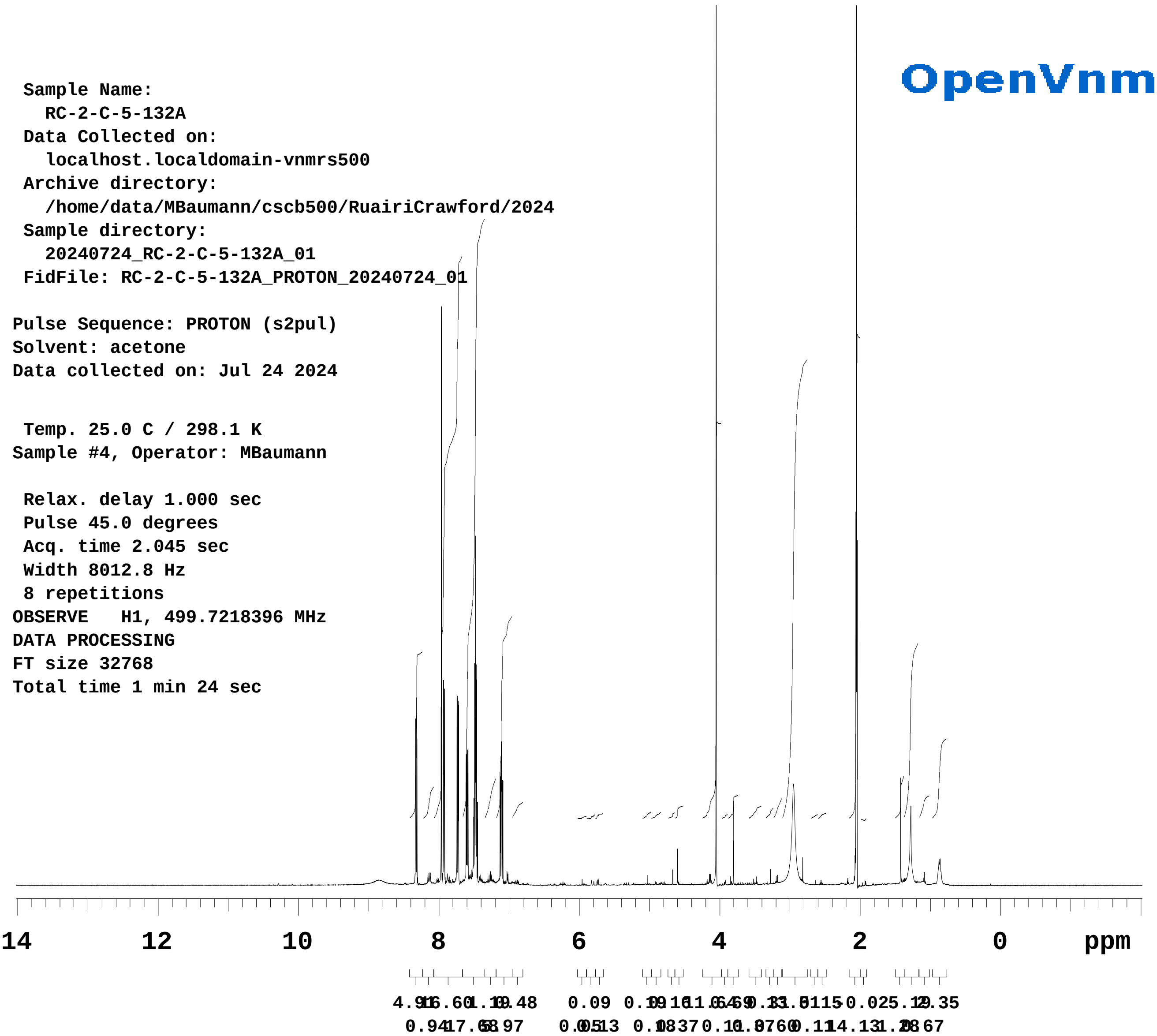

Plotname: RC-2-C-5-132A_PROTON_20240724_01_plot01
